# Haemorrhoidal artery ligation: Is Doppler guidance useful? A systematic review and meta‐analysis of randomized controlled trials

**DOI:** 10.1111/codi.70163

**Published:** 2025-07-15

**Authors:** Roberta Tutino, Arcangelo Picciariello, Mauro Santarelli, Veronica De Simone, Pierluigi Lobascio, Gianfranco Cocorullo, Marco Massani, Giusi Graziano, Giulio Aniello Santoro, Gaetano Gallo

**Affiliations:** ^1^ Department of General and Emergency Surgery AOU Città Della Salute e Della Scienza di Torino Torino Italy; ^2^ Department of Experimental Medicine University of Salento Lecce Italy; ^3^ National Institute of Gastroenterology S. De Bellis, IRCSS Research Hospital Castellana Grotte Italy; ^4^ Colorectal Surgery Unit, Faculty of Medicine and Surgery IRCSS San Raffaele Scientific Institute, Vita‐Salute University Milan Italy; ^5^ Department of Surgery University Hospital of Bari Bari Italy; ^6^ Department of Medicine of Precision and Regenerative and Jonic Area (DiMePRe‐J) University Hospital of Bari Bari Italy; ^7^ Department of Surgical, Oncological and Stomatological Disciplines University of Palermo Palermo Italy; ^8^ Division of General Surgery 1 Treviso Regional Hospital, AULSS 2 Marca Trevigiana Treviso Italy; ^9^ CORESEARCH, Center for Outcomes Research and Clinical Epidemiology Pescara Italy; ^10^ Third Referral Pelvic Floor Centre Treviso Regional Hospital, AULSS 2 Marca Trevigiana Treviso Italy; ^11^ Department of Surgery Sapienza University of Rome Rome Italy

**Keywords:** Doppler, haemorrhoid, haemorrhoidal artery ligation, haemorrhoidal vascularization, mucopexy

## Abstract

**Aim:**

Haemorrhoidal arterial ligation (HAL) with or without mucopexy (recto‐anal repair, RAR) is a minimally invasive technique for the treatment of haemorrhoidal disease. It is still debated if it should be performed with Doppler guidance (DG‐HAL) or without. The primary aim of this study was to find evidence that the use of Doppler guidance reduces the risk of recurrence. The secondary aim was to evaluate the benefit of Doppler guidance on postoperative complications.

**Methods:**

A comprehensive literature search was conducted according to the Preferred Reporting Items for Systematic Reviews and Meta‐Analyses (PRISMA) guidelines (2020). The population, intervention, comparison, outcomes and study design (PICOS) framework was used for eligibility criteria. Data were extracted independently by two reviewers for initial screening and for eligibility to be included in the meta‐analysis of randomized controlled trials. Random effects meta‐analysis, leave‐one‐out meta‐analysis and meta‐regression were performed to assess the main outcomes.

**Results:**

Seven randomized controlled trials, published between 2008 and 2023, were selected. They included 514 patients (mean age 49.13 years, 46% men) with different haemorrhoidal degrees. The HAL technique was performed in 255 patients and DG‐HAL in 259 patients. The meta‐analysis revealed that the DG‐HAL procedure was associated with a significant risk of recurrence (*P* = 0.007) and similar postoperative pain (*P* = 0.623) compared to the HAL procedure. In a subgroup analysis of patients treated with a combined RAR procedure, no differences were found between the DG‐HAL‐RAR and HAL‐RAR regarding the risk of recurrence (*P* = 0.36) and postoperative pain (*P* = 0.31).

**Conclusion:**

In conclusion, this meta‐analysis did not find superiority of DG‐HAL over HAL with or without RAR in reducing postoperative complications and recurrence.

**Key points**

- The use of Doppler guidance for selective haemorrhoidal artery ligation in the treatment of haemorrhoidal disease is debated.
- Our systematic review and meta‐analysis of randomized controlled trials failed to demonstrate the superiority of the Doppler‐guided procedure over the blind procedure in terms of recurrence and postoperative complications.

## INTRODUCTION

The pathophysiology of haemorrhoidal disease (HD) is still controversial between the vascular theory, in which hyperflow in the haemorrhoidal arteries plays the main role, and the sliding theory, which considers the damage to the supporting connective tissue with the downward displacement of the anal cushions as the key factor. In fact, these mechanisms could coexist and both play a role, as confirmed by the numerous surgical procedures introduced in the last 100 years [1, 2, 3].

In 1995, Morinaga et al. proposed selective ligation of the haemorrhoidal artery under Doppler guidance (DG‐HAL) to reduce the inflow, resulting in gradual shrinkage and fixation of the haemorrhoidal tissue [4]. Although this procedure was effective in controlling bleeding (88%–96%) [5, 6], a high recurrence rate (60%) was reported [7], mainly due to persistent prolapse.

Subsequent surgical procedures have attempted to combine selective ligation of the haemorrhoidal artery with ‘recto‐anal repair’ of the prolapsed mucosal tissue (DG‐HAL‐RAR) [8]. Since then, different techniques and devices have been described [9, 10, 11] with arterial ligation performed with or without DG and mucopexy performed below the point of artery closure [10] or separately from the ligation using running sutures to treat large, prolapsed components (Anolift) [11]. In particular, the benefit of DG has been debated since, although it allows accurate identification and ligation of the terminal branches of the superior rectal arteries, which almost exclusively provide the vascularization of the corpus cavernosum recti and distal rectum, the distal distribution of the superior rectal arteries is not limited to sectorial o'clock positions but is distributed circumferentially in each quadrant as arteries move from the perirectal fat toward the submucosa [12, 13, 14]. Consequently, artery ligation can be performed without DG, resulting in considerable cost savings [15].

The primary aim of this systematic review and meta‐analysis of randomized controlled trials (RCTs) was to find evidence that the use of DG effectively reduces the risk of recurrence. The secondary aim was to evaluate the benefit of DG on postoperative complications.

## MATERIALS AND METHODS

**FIGURE 1:**
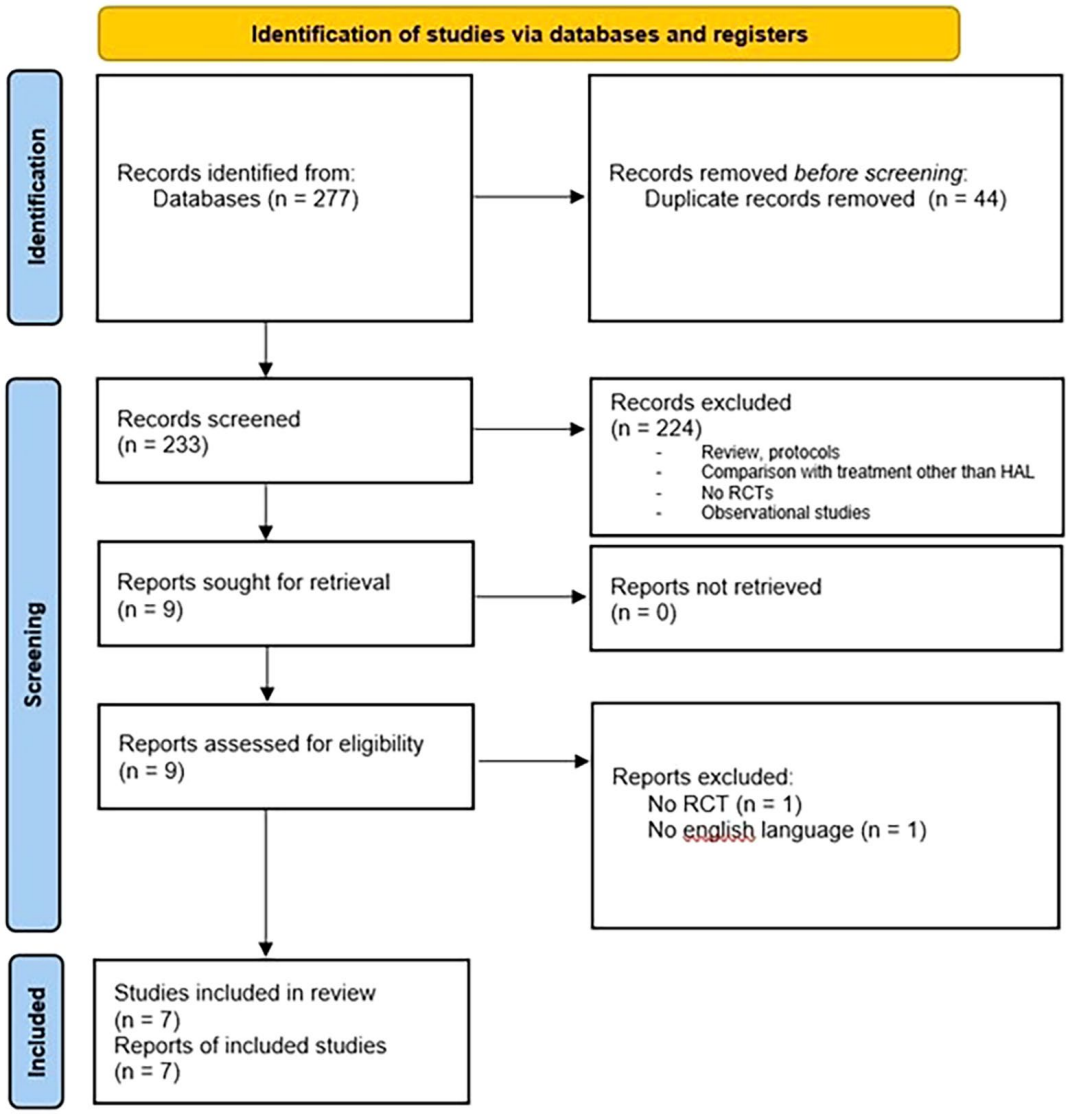
PRISMA diagram.

A systematic review and meta‐analysis of RCTs was conducted to compare HAL with DG‐HAL procedures. The combination of RAR and artery ligation was considered for a subgroup analysis. The selection process was undertaken in accordance with the 2020 Preferred Reporting Items for Systematic Reviews and Meta‐Analyses (PRISMA) guidelines [16] (Figure 1).

### Eligibility criteria

The population, intervention, comparison, outcomes and study design (PICOS) model was used: (1) patient/population—adult patients >18 years old suffering HD; (2) intervention—DG‐HAL with or without RAR; (3) comparison—HAL with or without RAR; (4) outcomes—recurrences and postoperative pain; (5) study design—RCTs. Data collected were (1) the indication for treatment (haemorrhoidal degree); (2) treatment modality; (3) post‐procedural complications; (4) outcome (symptoms' resolution rates); (5) follow‐up period. The literature search comprised all the inherent published original papers up to July 2024. English language papers were included. Exclusion criteria were non‐randomized trials, comparison with techniques other than HAL/DG‐HAL, haemorrhoidal degree and recurrences not reported.

### Information source and search strategy

Original papers were identified by search on PubMed/MEDLINE, the Cochrane Library, Scopus and Embase databases. The search strategy was based on the following search terms with the appropriate combinations: Haemorrhoids/surgery [Mesh] OR Haemorrhoids/therapy [Mesh] OR haemorrhoid*[Title/Abstract] AND RAR [Title/Abstract] OR dearterialization [Title/Abstract] OR THD [Title/Abstract] OR rect*‐an* repair* [Title/Abstract] OR transanal haemorrh* dearterializa* [Title/Abstract] OR mucopex* [Title/Abstract] OR Doppler [Title/Abstract] OR THD [Title/Abstract]. Reference lists of all full texts were hand‐selected for any additional studies.

### Study records

Open Science Framework (OSF) REGISTRIES, 7 June 2024, Registration DOI:

https://doi.org/10.17605/OSF.IO/F5KBE.

### Data process

The systematic processing of the data included the following. (1) Data management: an electronic record with an Excel framework was created including sample sizes and initial numbers. (2) Selection process: articles were searched by two independent reviewers for initial screening and for eligibility to be included in the meta‐analysis of RCTs. (3) Data collection process: data were extracted in a pilot form and then selected according to the missing or superfluous ones. Disagreements among reviewers were solved by discussion. (4) Data items: number of patients, patient demographical data, haemorrhoidal degree according to Goligher's classification, postoperative complications and recurrence rate at follow‐up were analysed. (5) Outcomes and prioritization: recurrences at follow‐up were considered as the primary outcome. The occurrence of postoperative complications (pain, bleeding, reoperation) represented the secondary outcome. (6) Risk of bias in individual studies: the risk of bias in RCTs was assessed using the Joanna Briggs Institute (JBI) critical appraisal tool [17], which provided a structured approach to evaluate study quality and potential sources of bias.

### Statistical analysis

The comparison between the two procedures was summarized in terms of relative risk (RR) for dichotomous outcomes and standardized mean difference (MD) for continuous outcomes with 95% CI. Heterogeneity between studies was evaluated with Cochran's *Q* and the *I* ^2^ statistics [18]. A subgroup analysis was included to investigate potential sources of heterogeneity on procedure effects. The DerSimonian and Laird random effect model was employed to pool data. To explore the impact of each study on the overall estimate and to identify influential studies a leave‐one‐out meta‐analysis was also performed. As a last step, a meta‐regression was considered to study the relationship between the different degrees of haemorrhoidal disease and effect estimate of the primary outcome. The results are shown in forest plots and tables generated, together with risk effect measures, by the software Comprehensive Meta‐Analysis Version 4 [19].

## RESULTS

### Study selection

A total of 277 records were screened, out of which 270 were excluded based on the eligibility criteria. Overall, seven RCTs comparing HAL and DG‐HAL with or without RAR [15, 20, 21, 22, 23, 24, 25] were selected for the analysis (Figure 1). The studies, published between 2008 and 2023, included a total of 514 patients (mean age 49.13 ± 12.97; men 46%), treated for HD. Along with degree III HD patients, one study also included degree I HD patients, two studies included degree II HD patients and two studies included degree IV HD patients.

### Techniques and devices

The HAL procedure was performed in 255 patients and DG‐HAL in 259 patients. The studies varied in terms of the devices used for artery ligation, mucopexy and the modality of Doppler. HAL‐Doppler II (AMI, Feldkirch, Austria) and Trilogy devices (AMI, Feldkirch, Austria) used pulsed Doppler, while KM‐25 (VaiDan Medical Corporation, St Petersburg, FL, USA), THD (transanal haemorrhoidal dearterialization) (THD America Inc., Tampa, FL, USA) and THD Slide™ (THD SpA, Correggio, Italy) devices used continuous‐wave Doppler. Number of ligations varied across the studies and across patients (Table 1).

**TABLE 1:** Patients' demographics and surgical techniques and devices used in the selected trials.

First author (year)	Period	Technique	No. of patients	Age (mean)	Sex M/F	HD degree	Anaesthesia	Device	No. of ligatures	No. of ligatures for RAR
Gupta (2011) [23]	ND	DG‐HAL‐RAR HAL‐RAR	23 22	44 ± 11.2 47 ± 10.4	13/11 12/12	III	General or spinal	KM‐25 (continuous‐ wave Doppler) No device	6–11 3	3 3
Schuurman (2012) [20]	2008–2010	DG‐HAL HAL	38 35	50 ± 13 51 ± 13.7	22/14 24/10	II–III	General	THD (continuous‐ wave Doppler) THD	6 6	0 0
Aigner (2015) [24]	2010–2013	DG‐HAL‐RAR HAL‐RAR	20 20	49 ± 12.6 57.5 ± 16	12/8 11/9	III	General	THD Slide (continuous‐ wave Doppler) THD Slide	ND ND	ND ND
Zhai (2016) [21]	2010–2011	DG‐HAL HAL‐RAR	50 50	50.6 ± 14 43.6 ± 15	16/34 23/27	III	General or spinal	HAL‐Doppler II (pulsed Doppler) Anoscope JINTAN	6 2–4	0 3
Huang (2016) [25]	2012–2013	DG‐HAL HAL	80 80	52 ± 12.6 53.2 ± 12	47/33 43/37	III–IV	Spinal	HAL Doppler II (pulsed Doppler) Anoscope JINTAN	ND ND	0 0
Perivoliotis (2020) [22]	ND	DG‐HAL‐RAR HAL‐RAR	30 30	52.9 ± 17 52.5 ± 18	17/13 18/12	I–II–III	Spinal Pudendal	ND ND	ND 3	ND ND
Alemrajabi (2023) [15]	2019–2020	DG‐HAL‐RAR HAL‐RAR	18 18	44.7 ± 7.3 40.9 ± 8.3	9/9 8/10	III–IV	ND	Trilogy (pulsed Doppler) Trilogy	Up to 6 Up to 6	4 4

Abbreviations: DG, Doppler guided; HAL, haemorrhoidal artery ligation; HD, haemorrhoidal disease; ND, not defined; RAR, recto‐anal repair; THD, transanal haemorrhoidal dearterialization.

### Quality assessment

**FIGURE 2:**
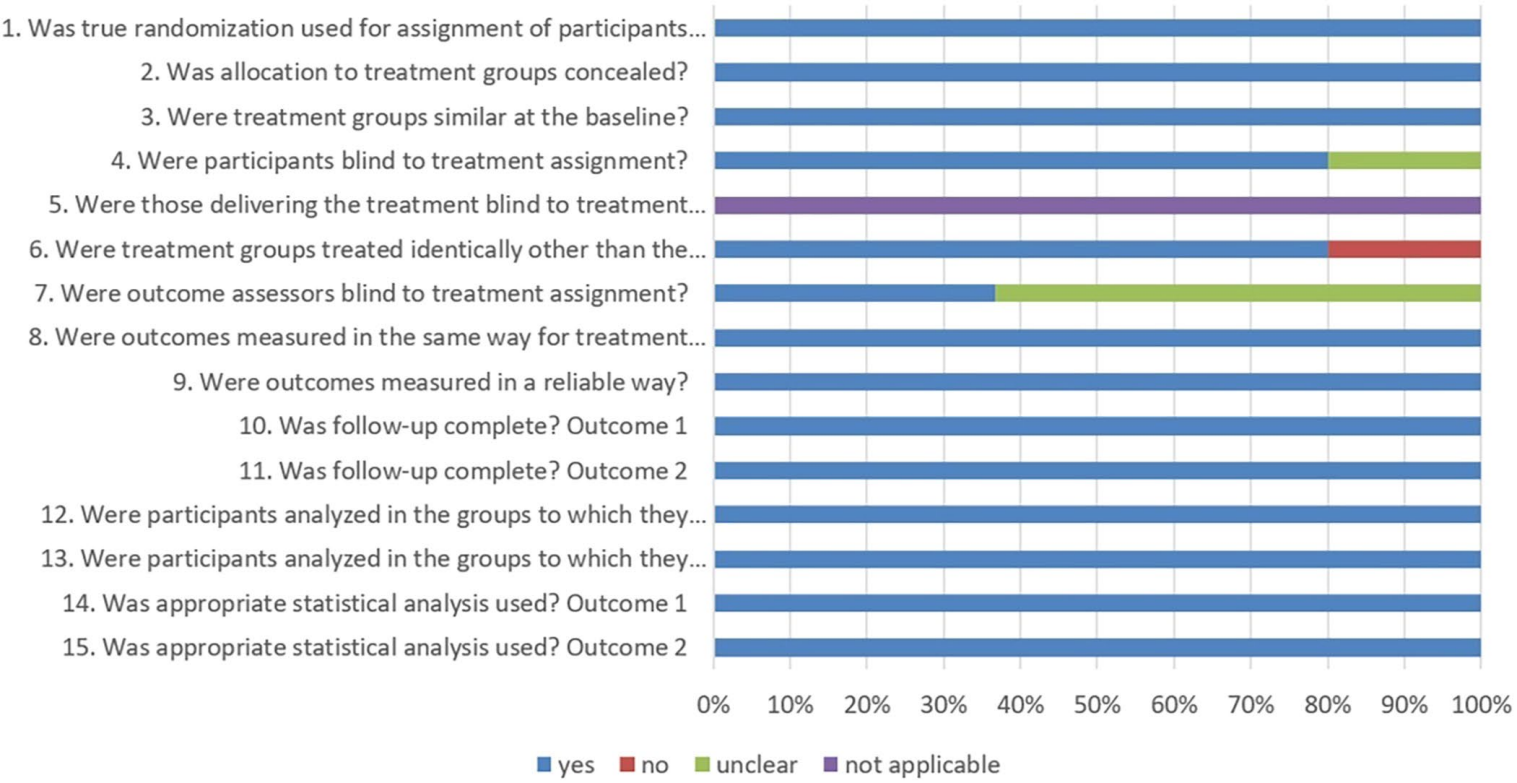
The revised Joanna Briggs Institute (JBI) critical appraisal tool for the assessment of risk of bias for randomized controlled trials.

Based on the revised JBI critical appraisal tool for the assessment of risk of bias for RCTs, the quality of the included studies was good (Figure 2). True randomization and allocation concealment was adequately described in all trials. Treatment groups were similar at baseline. Outcome assessor blinding was declared in three studies [20, 21, 22], but was unclear in four studies [19, 23, 24, 25]. In all trials analysed, the minimum follow‐up was 6 months, and the measurement of reported outcomes was reliable.

### Studies’ summary

The patients' demographics, techniques and devices used are summarized in Table 1 and the operative outcomes in Table 2.

**TABLE 2:** Surgical outcomes of the selected trials.

First author (year)	Study type	Technique	No. of patients	HD degree	Postoperative pain	Postoperative bleeding	Follow‐up (months)	Recurrences
Gupta (2011) [23]	RCT	DG‐HAL‐RAR HAL‐RAR	23 22	III	4.4 (2.9–6.3) 2.2 (1.7–5.9)	1 (4.4%) 0	12 12	3 (13%) 4 (18%)
Schuurman (2012) [20]	RCT	DG‐HAL HAL	38 35	II–III	3.9 ± 1.0 4.5 ± 1.0	3 (7.9%) 0	6 6	6 (17%) 3 (8%)
Aigner (2015) [24]	RCT	DG‐HAL‐RAR HAL‐RAR	20 20	III	0.1 ± 0.2 0.2 ± 0.4	ND ND	12 12	2 (10%) 1 (5%)
Zhai (2016) [21]	RCT	DG‐HAL HAL‐RAR	50 50	III	0.8 ± 1.4 1.0 ± 1.2	1 (2%) 2 (4%)	24 24	21 (42.8%) 4 (9.3%)
Huang (2016) [25]	RCT	DG‐HAL HAL	80 80	III–IV	30 mild, 6 moderate, 4 severe (NS) 32 mild, 9 moderate, 2 severe (NS)	12 (15%) 10 (12.5%)	12 12	18 (22.5%) 2 (2.5%)
Perivoliotis (2020) [22]	RCT	DG‐HAL‐RAR HAL‐RAR	30 30	I–II–III	4.53 ± 2.34 1.6 ± 1.8	5 (16.7%) 1 (3.3%)	12 12	15 (50%) 5 (16.7%)
Alemrajabi (2023) [15]	RCT	DG‐HAL‐RAR HAL‐RAR	18 18	III–IV	6.7 ± 1.5 7.06 ± 1.58	ND ND	6 6	1 (5.6%) 2 (11.1%)

Abbreviations: DG, Doppler guided; HAL, haemorrhoidal artery ligation; HD, haemorrhoidal disease; NS, not significant; RAR, recto‐anal repair; RCT, randomized controlled trial.

Gupta et al. [23] assessed the benefits of DG‐HAL‐RAR versus HAL‐RAR reporting no significant difference in recurrence rates at 1‐year follow‐up, but higher postoperative pain in the DG group. Schuurman et al. [20] compared the outcomes of HAL/THD procedures with and without DG. Postoperative pain did not differ significantly; however, at the 6‐month follow‐up, 17% of patients in the DG‐HAL group (vs. 8%) considered themselves to need subsequent treatment. Aigner et al. [24] reported less postoperative pain in the DG‐HAL versus the mucopexy group during the first week and no significant difference in recurrence rates at the 12‐month follow‐up. Huang et al. [25] found no difference in postoperative pain between DG‐HAL versus HAL; however, at the 1‐year follow‐up, recurrence rate was higher in the DG‐HAL group. No difference in postoperative pain was reported by Zhai et al. [21], who compared DG‐HAL without mucopexy versus suture‐fixation mucopexy. At 24 months, the recurrence rates for prolapsing haemorrhoids or bleeding was lower in the mucopexy group. Perivoliotis et al. [22] reported higher remission rate and lower morbidity, postoperative pain and recurrence rate in the HAL‐haemorrhoidopexy group versus the DG‐HAL group. Alemrajabi et al. [15] found no significant difference between DG‐HAL‐RAR and HAL‐RAR in terms of postoperative pain and complications and recurrence rate.

Haemorrhoidal grade was not a risk factor for recurrence in the RCT by Alemrajabi et al. [15] and Schuurman et al. [20] regardless of the surgical method. However, in the first trial recurrences were related to the Wexner score and body mass index in both treatment groups. Perivoliotis et al. [22] reported that the American Society of Anesthesiologists score was associated with higher recurrence rate.

## META‐ANALYSIS OF THE INCLUDED STUDIES

### Recurrences

**FIGURE 3:**
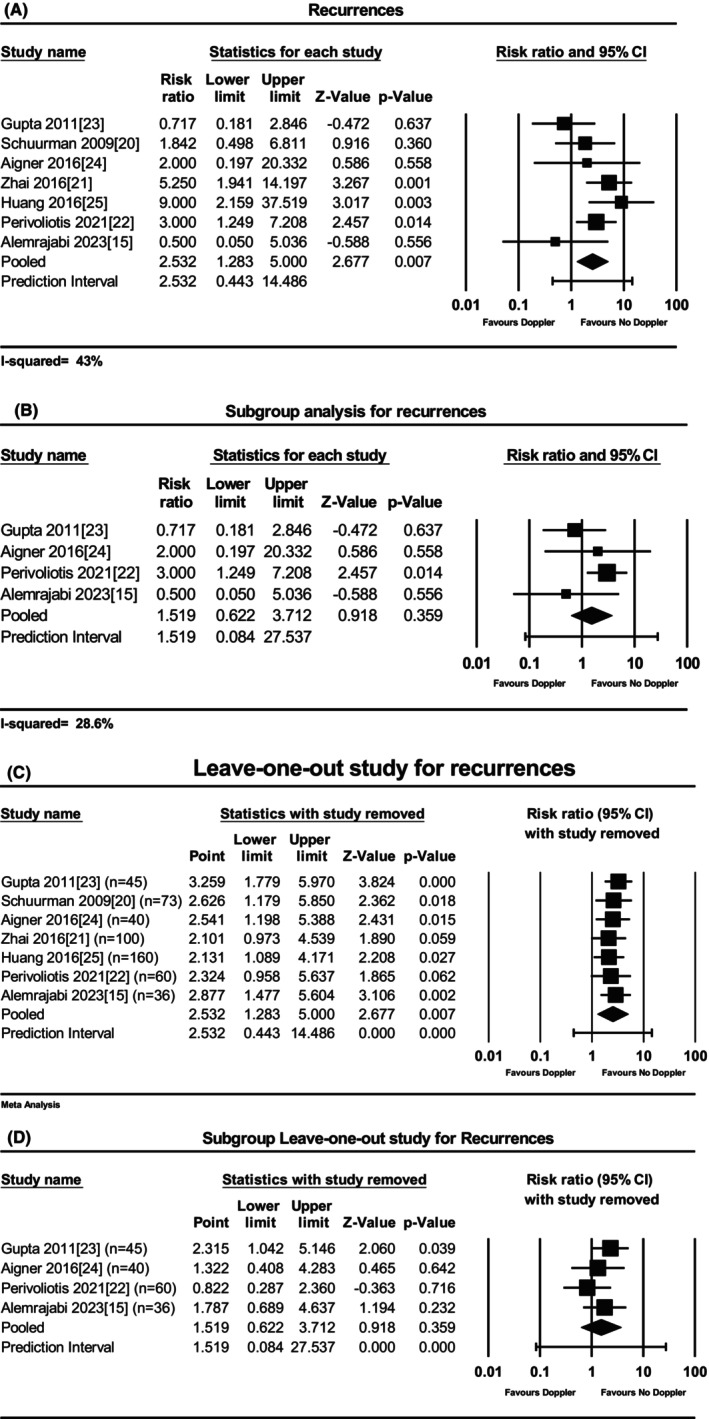
Forest plot for recurrences: (A) overall (*Q* = 10.49; *I* ^2^ = 43%); (B) subgroup analysis in HAL‐RAR group (*Q* = 0.78, *I* ^2^ = 0%); (C) leave‐one‐out analysis; (D) subgroup leave‐one‐out analysis (see text).

The DG‐HAL procedure was overall related to increased risk of recurrence compared to the HAL procedure (seven studies, 514 participants, RR 2.53, 95% CI 1.28–5.00, *P* = 0.007). Heterogeneity was low/moderate (*I* ^2^ = 43%) (Figure 3A). A subgroup meta‐analysis of studies combining mucopexy and artery ligation showed no significant differences in recurrence risk between the DG‐HAL‐RAR and HAL‐RAR groups (four studies [15, 22, 23, 24], 181 participants, RR 1.52, 95% CI 0.08–27.54, *P* = 0.36, no significant heterogeneity: *I* ^2^ = 28.62% (low) (Figure 3B). Figure 3C illustrates the results of the leave‐one‐out meta‐analysis, where each study was sequentially excluded to assess its individual influence on the overall pooled effect estimate. The exclusion of the study of Gupta et al. [23] or Schuurman et al. [20] or Alemrajabi et al. [15] revealed a larger impact on the overall risk ratio; in particular omitting the first study caused the pooled estimate to increase by roughly 0.8. The impact of the study of Gupta et al. [23] emerged also in the subgroup analysis where its exclusion resulted in a significant increased overall risk of recurrence in DG‐HAL (Figure 3D).

### Postoperative pain

**FIGURE 4:**
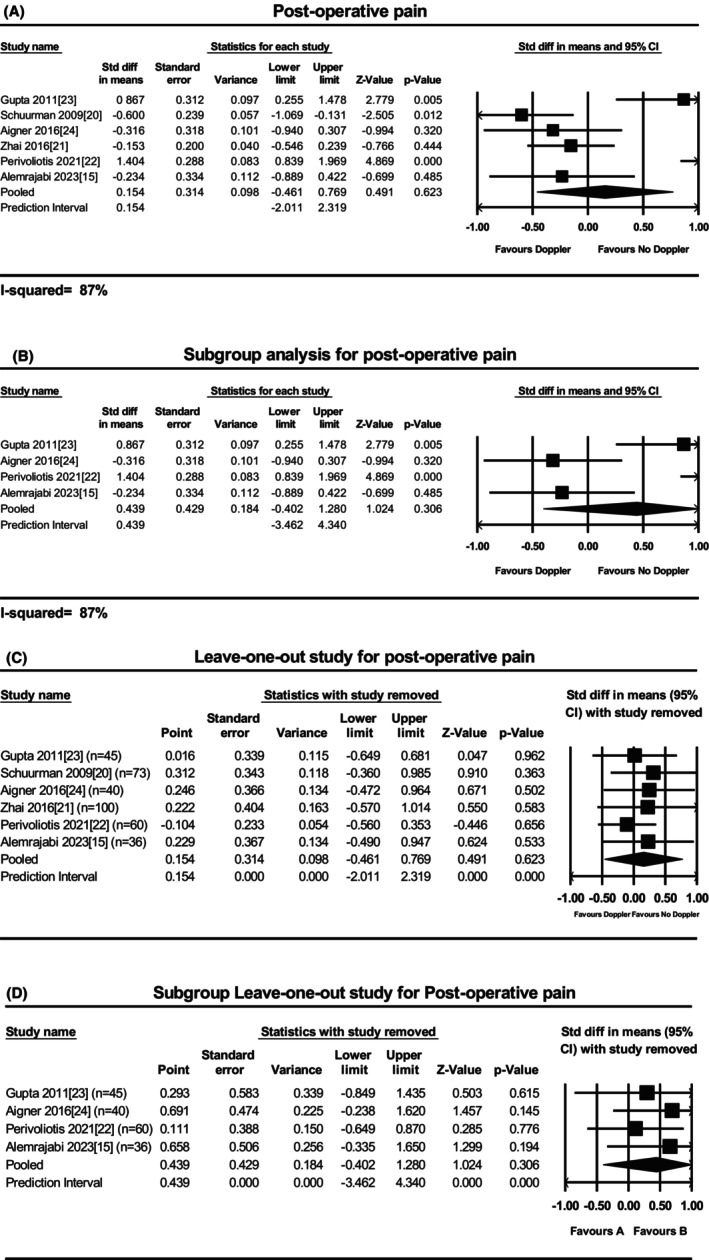
Forest plot for postoperative pain: (A) overall (*P* = 0.623; *I* ^2 =^ 87%); (B) subgroup analysis in HAL‐RAR group (*P* = 0.306; *I* ^2^ = 87%); (C) leave‐one‐out analysis; (D) subgroup leave‐one‐out analysis (see text).

Overall, no significant reduction in postoperative pain in terms of median visual analog scale was reported with the use of Doppler (six studies [15, 20, 21, 22, 23, 24], 354 participants, MD 0.15, 95% CI −0.46 to 0.77, *P* = 0.623). Heterogeneity was high (*I* ^2^ = 87%) (Figure 4A). In the subgroup meta‐analysis of studies combining RAR and HAL, no significant differences were found in postoperative pain between the DG group and the blinded group (four studies [15, 22, 23, 24], 181 participants, MD 0.44, 95% CI −0.40 to 1.28, *P* = 0.31; high heterogeneity: *I* ^2^ = 87%) (Figure 4B). The sequential exclusion of each study in the leave‐one‐out meta‐analysis did not show significant influence on the pooled estimate, neither in the overall nor in the subgroup analysis (Figure 4C, D).

### Postoperative bleeding

**FIGURE 5:**
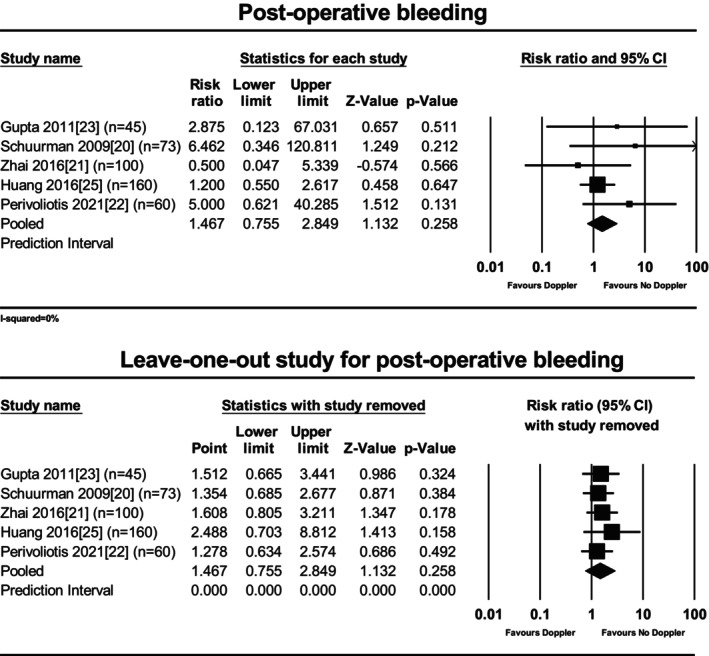
Forest plot for postoperative bleeding: (A) overall (*P* = 0.258; *I* ^2^ = 0%); (B) leave‐one analysis.

No significant reduction in postoperative bleeding was reported with the use of Doppler (five studies [20, 21, 22, 23, 25], 438 participants, RR 1.47, 95% CI 0.75–2.85, *P* = 0.26). Heterogeneity was low (*I* ^2^ = 0%) (Figure 5A). The subgroup meta‐analysis was not performed due to the unreported data in some of the relative studies. No significant results emerged from the leave‐one‐out meta‐analysis (Figure 5B).

### Meta‐regression between degrees of HD and recurrences

The studies were classified based on haemorrhoidal degrees of the included patients (less than, greater than or equal to degree III). In accordance with the test of the model, we found that the covariate haemorrhoidal degree was not related to the effect estimate and did not affect the risk of recurrence (*P* = 0.95). The results of meta‐regression are summarized in Table 3.

**TABLE 3:** Meta‐regression to study the relationship between the different grades of haemorrhoidal disease and effect estimate of primary outcome.

Covariate	Coefficient	Standard error	95% lower	95% upper	Z value	Two‐sided *P* value
Intercept	0.75	0.67	−0.57	20.67	1.12	0.26
<Grade III	0.13	0.99	−18.22	20.85	0.13	0.89
>Grade III	0.36	11.25	−18.46	25.64	0.32	0.75
**Statistics for model 1**						
Test of the model: *Q* = 0.10, df = 2, *P* = 0.9503						
Goodness of fit: tau^2^ = 0.7755, tau = 0.8806, *I* ^2^ = 60.16%, *Q* = 10.04, df = 4, *P* = 0.0398						

*Note*: Main results for model 1, random effects (MM), Z distribution, log risk ratio.

## DISCUSSION

This systematic review and meta‐analysis of seven RCTs found insufficient evidence that the use of DG for HAL, with or without RAR, offers significant advantage in terms of recurrence and postoperative complications. The analysis of 514 patients showed an overall lower recurrence rate in the no‐DG treatment groups (RR 2.53, 95% CI 1.28–5.00, *P* = 0.007). However, the heterogeneity between the studies was from low to moderate (*Q* ^2^ = 10.49, *P* = 0.11; *I* ^2^ = 43%), probably due to the different modalities of performing DG‐HAL and to the combination or not with RAR. The results are confirmed by a leave‐one‐out meta‐analysis where the exclusion of the studies of Gupta, Schuurman or Alemrajabi revealed a larger impact on the overall risk ratio. Since some studies analysed patients with different haemorrhoidal degrees, we conducted a meta‐regression that showed that this covariate did not affect the risk of recurrences.

This meta‐analysis also confirmed that the procedure limited to the ligation of the haemorrhoidal artery, even when performed under DG, is an insufficient treatment for HD and does not provide effective long‐term results. Schuurman et al. [20] hypothesized that to achieve a beneficial effect the ligation of the main artery is not necessary or the use of a Doppler modality. Aigner et al. [24] reported that repositioning of the anal transitional zone was the key step in patients with HD who reported satisfactory benefits. This was confirmed by Zhai et al. [21] who showed that mucopexy was more effective than DG‐HAL to reduce the recurrence rate (9.3% vs. 42.8%, respectively).

In the present meta‐analysis, in the subgroup of patients treated by HAL‐RAR, the use of DG had no significant advantages on postoperative recurrence [RR 0.82, 95% CI 0.29–2.36, *P* = 0.716 with low heterogeneity among the studies (*I* ^2^ = 0%)] or postoperative pain [standardized difference in means 0.11, 95% CI 0.65–0.87, *P* = 0.776, although in the presence of high heterogeneity (*I* ^2^ = 77%). These results are consistent with the overall main analysis on postoperative pain that demonstrated similar results between the DG or no‐DG groups (standardized difference in means 0.15, 95% CI 0.46–0.77, *P* = 0.623). The Doppler localization of the haemorrhoidal vessels did not contribute to improve the clinical outcome. Alemrajabi et al. [15] suggested that simple mucopexy and RAR with HAL at standard zones (1, 5, 7 and 11 o'clock positions) was as effective as DG‐HAL for the treatment of HD degrees III and IV in terms of recurrence rate and postoperative complications, but less expensive. Huang et al. [25] and Perivoliotis et al. [22] also found fewer postoperative complications and recurrences respectively in the HAL group and HAL‐RAR group.

In a systematic review by Pucher et al. [7], the overall recurrence rate of the DG‐HAL procedure was 17.5%. Giordano et al. [26] reported a recurrence rate of 10.8% for prolapse and 9.7% for bleeding at 1‐year follow‐up in a systematic review on DG‐HAL performed by using the THD device combined or not with RAR. In 2023, Fathallah et al. [27] found a higher recurrence rate (40%) at 5 years after DG‐HAL‐RAR with THD. In their study patients’ age and the presence of both bleeding and prolapse at the time of surgery were significant predictors of recurrence (*P* = 0.012 and *P* < 0.001). A cohort study by Bonomo et al. [28] comparing HAL‐RAR with or without DG reported an overall recurrence rate of 25.4% (23.7% for DG‐HAL and 28.3% for HAL) at long‐term follow‐up (>3 years in 59% of cases). Popovtsev et al. [29] reported comparable results in terms of pain (*P* = 0.24), incidence of postoperative complications (*P* = 0.51) and recurrences (*P* = 0.31) between blind or DG‐HAL combined with mucopexy for the treatment of degree II–III HD.

This meta‐analysis has some limitations. Although we included only RCTs, the analysed studies were heterogeneous in terms of haemorrhoidal degrees, techniques of artery ligation and RAR and type of devices used. Three RTCs did not declare a blinded process in evaluation of the outcomes. Another limitation was the small sample size of some trials.

In conclusion, this meta‐analysis did not find superiority of DG‐HAL over HAL with or without RAR in reducing postoperative complications and recurrence. Mucopexy was the key factor to reduce the recurrence rate of these procedures. The ligation of the haemorrhoidal artery, even when selectively performed under DG, did not provide effective long‐term results if not associated with RAR. Larger RCTs are needed to confirm the clinical implications of our findings.

## AUTHOR CONTRIBUTIONS

**Roberta Tutino:** Conceptualization; data curation; methodology; validation; writing – original draft; writing – review and editing. **Arcangelo Picciariello:** Methodology; validation. **Mauro Santarelli:** Data curation; validation. **Veronica De Simone:** Data curation; validation. **Pierluigi Lobascio:** Conceptualization; visualization. **Gianfranco Cocorullo:** Supervision. **Marco Massani:** Supervision. **Giusi Graziano:** Methodology; formal analysis. **Giulio Aniello Santoro:** Methodology; formal analysis; writing – review and editing. **Gaetano Gallo:** Methodology; investigation; writing – review and editing.

## FUNDING INFORMATION

None.

## CONFLICT OF INTEREST STATEMENT

The authors declare no conflicts of interest.

## ETHICS STATEMENT

Not required.

## REGISTRATION

Open Science Framework (OSF) REGISTRIES, 7 June 2024, Registration DOI: https://doi.org/10.17605/OSF.IO/F5KBE.

## Data Availability

The data that support the findings of this study are available from the corresponding author upon reasonable request.
